# Nonlinear Fokker–Planck Equation Approach to Systems of Interacting Particles: Thermostatistical Features Related to the Range of the Interactions

**DOI:** 10.3390/e22020163

**Published:** 2020-01-31

**Authors:** Angel R. Plastino, Roseli S. Wedemann

**Affiliations:** 1 CeBio y Departamento de Ciencias Básicas, Universidad Nacional del Noroeste de la Província de Buenos Aires, UNNOBA, Conicet, Roque Saenz Peña 456, Junin 6000, Argentina; 2 Instituto de Matemática e Estatística, Universidade do Estado do Rio de Janeiro, Rua São Francisco Xavier, 524, Rio de Janeiro 20550-900, RJ, Brazil; roseli@ime.uerj.br

**Keywords:** nonlinear Fokker–Planck equations, *S_q_* entropies, long-range interactions, gravitation, 05.90.+m

## Abstract

Nonlinear Fokker–Planck equations (NLFPEs) constitute useful effective descriptions of some interacting many-body systems. Important instances of these nonlinear evolution equations are closely related to the thermostatistics based on the $S_{q}$ power-law entropic functionals. Most applications of the connection between the NLFPE and the $S_{q}$ entropies have focused on systems interacting through short-range forces. In the present contribution we re-visit the NLFPE approach to interacting systems in order to clarify the role played by the range of the interactions, and to explore the possibility of developing similar treatments for systems with long-range interactions, such as those corresponding to Newtonian gravitation. In particular, we consider a system of particles interacting via forces following the inverse square law and performing overdamped motion, that is described by a density obeying an integro-differential evolution equation that admits exact time-dependent solutions of the *q*-Gaussian form. These *q*-Gaussian solutions, which constitute a signature of $S_{q}$-thermostatistics, evolve in a similar but not identical way to the solutions of an appropriate nonlinear, power-law Fokker–Planck equation.

## 1. Introduction

The thermostatistics derived from the $S_{q}$ non-additive entropies [1,2,3] exhibit interesting links with the statistical properties of (i) systems with long-range interactions, including cases of self-gravitating systems; and (ii) systems described by nonlinear, power-law diffusion or Fokker–Planck equations. In this paper, we revisit some aspects of these two areas of application of the $S_{q}$-thermostatistics, with the aim of exploring possible connections between them.

The statistical physics of systems with long-range interactions and, in particular, of self-gravitating systems, was the first promising area of application of the $S_{q}$-thermostatistics that was identified. This line of enquiry started with the discovery of the close connection between the $S_{q}$-thermostatistics and stellar polytropic distributions, which provided the first hint pointing towards concrete applications of the $S_{q}$-statistics [4]. The polytropic distributions, which play a distinguished role in theoretical astrophysics [5]—particularly in connection with the study of self-gravitating systems like galaxies—turned out to be distributions that optimize the $S_{q}$ entropies under the constraints imposed by the total mass and the total energy of the system [4]. The polytropic distributions are distributions in position–velocity space that are exact solutions of the coupled Vlasov–Poisson equations. These distributions are *q*-exponentials of the energy per particle. At a given spatial point, the associated velocity distribution is a *q*-Gaussian. The discovery of the connection between the polytropic distributions and the $S_{q}$ entropies suggested that the $S_{q}$-thermostatistics might be relevant for the study of the thermodynamical properties of systems with long-range interactions. Subsequent research generated a growing body of evidence attesting to that (see [1,2,6] and references therein). Indeed, the application of the $S_{q}$-thermostatistics to the study of the thermodynamics of various systems with long-range interactions has been, over the years, one of the main venues of research related to the $S_{q}$ entropies. In particular, the $S_{q}$-thermostatistics has been applied to the study of fundamental issues in self-gravitating systems, such as Jeans’ instability [7], relaxation processes [8], the relation between the dynamics and thermodynamics of these systems [9], and the problem of dark matter [10,11,12]. In the latter case, there is observational evidence that the $S_{q}$-statistics describes aspects of the dark matter haloes of galaxies [10,11,12]. Another of the most fruitful applications of the $S_{q}$-thermostatistics to physics (and other scientific disciplines) is based on its connection with nonlinear diffusion and Fokker–Planck equations, which was first pointed out in [13]. Nonlinear Fokker–Planck equations govern the behavior of a wide family of systems in physics, biology, and other branches of science [13,14,15,16,17,18,19,20,21,22,23,24,25,26,27,28]. Nonlinear diffusion processes [29,30] are important in various fields, such as in the study of the spread of biological populations [31,32]. They have also been used to model the spread of energy in nonlinear lattices [33]. The nonlinear diffusion and Fokker–Planck equations are also closely related to nonlinear versions of the Schroedinger, Dirac, and Klein–Gordon equations [34] admitting complex, *q*-plane wave, soliton-like, analytical solutions that comply with the Einstein–Planck–de Broglie relations [34], and to other nonlinear wave equations related to the $S_{q}$-thermostatistics, having either real *q*-Gaussian solutions or exponential plane wave solutions modulated by *q*-Gaussians [35].

An important class of systems described by nonlinear Fokker–Planck equations is given by confined many-body systems with short-range interactions, in the regime of overdamped motion [20,21,22,23,24,25]. This class of systems includes, as a prominent and interesting example, systems of interacting vortices in type-II superconductors [20,21]. On the one hand, the $S_{q}$-thermostatistics constitutes a useful theoretical instrument for studying thermodynamical aspects of systems with long-range interactions. On the other hand, one of the most developed applications of the $S_{q}$-thermostatistics is related to the description of thermodynamical features of confined, overdamped systems with short-range interactions. Thus, two of the most successful areas of application of the $S_{q}$-statistics involve completely different types of systems. Our aim was to explore a possible connection between these two apparently detached fields of application of the $S_{q}$-statistics. We are going to study a particular instance of a system with long-range interactions that, as Newtonian gravitation, follows the inverse square law. The dynamics of this system has some similarities with systems governed by power-law nonlinear Fokker–Planck equations. In particular, we are going to show that this system admits exact, time-dependent solutions of the *q*-Gaussian form. The *q*-Gaussian densities are central to the $S_{q}$-thermostatistics and to its diverse applications [1,2,36,37,38,39]. The *q*-Gaussians are *q*-exponentials having an argument that is quadratic in the spatial or phase-space variables of the system under consideration. The q→1 limit of the *q*-Gaussians corresponds to the standard exponential Gaussian functions. There is a wide variety of natural phenomena where *q*-Gaussians are observed. It is therefore of considerable importance to identify and investigate dynamical mechanisms that may lead to *q*-Gaussians.

This paper is organized as follows. In Section 2 , we briefly review some basic aspects of the $S_{q}$-thermostatistical formalism. In Section 3, we re-visit the nonlinear Fokker–Planck approach to the dynamics of the overdamped motion of interacting particles, with special emphasis on the role played by the range of the interactions. In Section 4, we analyze a system of particles performing overdamped motion and interacting via long-range forces, described by an evolution equation admitting exact time-dependent *q*-Gaussian solutions, similar (but not identical) to those corresponding to a related power-law nonlinear Fokker–Planck equation. Finally, some conclusions are given in Section 5.

## 2. $S_{q}$ Entropies, q-Exponentials, and q-Gaussians

The $S_{q}$-thermostatistics is based on the non-additive power-law entropy $S_{q}\left[\sigma \right]$ [2] defined as

(1) $$S_{q}\left[\sigma \right]\;=\;\frac{k}{q-1}\int \sigma \left[1-{\left(\frac{\sigma }{\sigma_{0}}\right)}^{q-1}\right]d^{N}x\;,$$

where *k* is a constant determining the dimensions and units in which entropy is measured, *q* is a real parameter, and σ(x) is a probability density defined in an *N*-dimensional space. The constant $\sigma_{0}$ has the same dimensions as the density σ, x denotes a point in the space under consideration, and $d^{N}x$ is the *N*-dimensional volume element. In the limit q→1 the standard Boltzmann–Gibbs entropy, $S_{1}=-k\int \sigma \ln\left(\sigma /\sigma_{0}\right)d^{N}x$, is recovered. The power-law entropy $S_{q}$ constitutes a distinguished member among the family of generalized entropies that are currently being intensively studied [40,41,42].

An essential ingredient of the $S_{q}$ thermostatistical formalism is given by the *q*-exponential function. This function arises in connection with the constrained optimization of the entropic functional $S_{q}$ [1,2,43]. The *q*-exponential function is defined as

(2) $$\exp_{q}\left(x\right)=\left\{\begin{array}{cc} {\left[1+\left(1-q\right)x\right]}^{\frac{1}{1-q}}\;, & \mathrm{if}\;1+(1-q)x>0\;,\\ 0\;, & \mathrm{if}\;1+(1-q)x\leq 0\;. \end{array}\right.$$

In this work we use an alternative notation for the *q*-exponentials, given by $\exp_{q}\left(x\right)={\left[1+\left(1-q\right)x\right]}_{+}^{\frac{1}{1-q}}$.

Especially relevant are the *q*-Gaussian densities, which are proportional to *q*-exponentials with an argument quadratic in the space or phase-space variables characterizing the problem under consideration. For example, a one-dimensional *q*-Gaussian is proportional to $\exp_{q}\left[-\beta x^{2}\right]={\left[1-\left(1-q\right)\beta x^{2}\right]}_{+}^{\frac{1}{1-q}}$, where β is a real, positive parameter associated with the width of the *q*-Gaussian. These distributions appear in the study of diverse systems and processes in physics, biology, and other disciplines [1,2,36,37,38,39]. They constitute solutions—both stationary and time-dependent—of some nonlinear evolution equations of mathematical physics. The first evolution equation for which a connection with the $S_{q}$ entropy was discovered was the nonlinear Fokker–Planck equation with a power-law nonlinearity [13]. In one spatial dimension, the nonlinear Fokker–Planck equation is

(3) $$\frac{\partial \sigma }{\partial t}=D\frac{\partial^{2}}{\partial x^{2}}\left[\sigma {\left(\frac{\sigma }{\sigma_{0}}\right)}^{1-q}\right]+\frac{\partial }{\partial x}\left(\sigma \frac{dV}{dx}\right)\;,$$

where σ(x,t) is a time-dependent density, $\sigma_{0}$ is a constant with the same dimensions as σ, *D* is a diffusion constant, and V(x) is a potential function. For quadratic potentials, the power-law nonlinear Fokker–Planck equation admits exact time-dependent solutions with a *q*-Gaussian shape. The connection between this evolution equation and the $S_{q}$ entropy stimulated the development of a fruitful research field. Over the years, it has been extended in various directions, and applied to diverse problems in physics and elsewhere [13,14,15,16,17,18,19,20].

## 3. $S_{q}$ Thermostatistics of Overdamped Motion

Now, we briefly review the application of the power-law nonlinear Fokker–Planck equation to the thermostatistics of systems of confined particles interacting through short-range forces and moving in the overdamped regime [20,21,22,23,24,25]. We pay special attention to the kind of inter-particle forces for which this type of treatment can be implemented.

Let us consider a system of interacting particles (in an *N*-dimensional space) moving under the effects of an external confining potential as well as drag forces. The force acting on a test particle of mass *m* has three components: the force $F_{\mathrm{int}}$ due to the interaction with the other particles, the force −∇W due to the external confining potential *W*, and the drag force $-\alpha \frac{dx}{dt}$, where α is a positive constant. The test particle obeys the equation of motion

(4) $$m\frac{d^{2}x}{dt^{2}}=F_{\mathrm{int}}-\nabla W-\alpha \frac{dx}{dt}.$$

In the limit of overdamped motion, where the inertial effects are negligible, the equation of motion reduces to

(5) $$\frac{dx}{dt}=\frac{1}{\alpha }\left(F_{\mathrm{int}}-\nabla W\right).$$

For short-range inter-particle forces under appropriate conditions, the force $F_{\mathrm{int}}$ can be approximated as

(6) $$F_{\mathrm{int}}=-D\nabla \sigma ,$$

where the constant D depends only on the properties of the inter-particle force. Using (6), the equation of motion of a test particle becomes

(7) $$\frac{dx}{dt}=-\frac{1}{\alpha }\left(D\nabla \sigma +\nabla W\right).$$

The continuity equation governing the evolution of the spatial density σ(x,t) of a system of particles obeying (7) is

(8) $$\frac{\partial \sigma }{\partial t}=\frac{D}{\alpha }\nabla \cdot \left[\sigma \nabla \sigma \right]+\frac{1}{\alpha }\nabla \cdot \left[\sigma \nabla W\right].$$

The above evolution equation can be re-cast under the guise of a nonlinear Fokker–Planck equation:

(9) $$\frac{\partial \sigma }{\partial t}=D\nabla^{2}\left[\sigma \left(\frac{\sigma }{\sigma_{0}}\right)\right]+\nabla \cdot \left[\sigma \nabla V\right],$$

where

$$\begin{array}{ccc} \frac{D}{\sigma_{0}} & = & \frac{D}{2\alpha } \end{array}$$

(10) $$\begin{array}{ccc} V(x) & = & \frac{1}{\alpha }W\left(x\right). \end{array}$$

Let us now discuss in more detail the derivation of expression (6), for the force on a test particle resulting from its interaction with the other particles of the system. For short-range repulsive forces, one can assume that the particle interacts only with particles situated in its immediate neighborhood [20]. The (repulsive) force on a particle located at x due to a particle at $x^{\prime }$ is $-F\left(|r|\right)\frac{r}{\left|r\right|}$, with $r=x^{\prime }-x$. We assume that the strength F(|r|)≥0 of this force is a smooth function of $r=|x^{\prime }-x|$ that decreases with *r* quickly enough for the integral $\int_{0}^{\infty }r^{N}F\left(r\right)dr$ to converge. For short-range forces, one can make the approximation that, within the range of *r*-values (i.e., range of distances) for which F(r) differs appreciably from zero, the particle density σ(x) is well represented by the first-order expansion $\sigma \left(x^{\prime }\right)=\sigma \left(x\right)+\left(x^{\prime }-x\right)\cdot \left(\nabla \sigma \right)$ (for a detailed discussion of this approximation see [20]). The force $F_{\mathrm{int}}\left(x\right)$ on a particle at x, due to its interaction with the other particles of the system, can be expressed as:

(11) $$\begin{array}{ccc} F_{\mathrm{int}}\left(x\right) & = & -\int \sigma \left(x^{\prime }\right)\;F(|x^{\prime }-x\left|\right)\;\frac{\left(x^{\prime }-x\right)}{|x^{\prime }-x|}\;d^{N}x^{\prime }\\ & = & -\int \;\left[\sigma \left(x\right)+\left(x^{\prime }-x\right)\cdot \left(\nabla \sigma \right)\right]\;F(|x^{\prime }-x\left|\right)\;\frac{\left(x^{\prime }-x\right)}{|x^{\prime }-x|}\;d^{N}x^{\prime }\\ & = & -\int \;\left[r\cdot (\nabla \sigma )\right]\;\left(\frac{F(r)}{r}\right)\;r\;d^{N}r\\ & = & -\int \;\left[\sum_{i,j=1}^{N}\;y_{i}y_{j}{\left(\nabla \sigma \right)}_{i}{\hat{y}}_{j}\right]\;\left(\frac{F(r)}{r}\right)\;d^{N}r\\ & = & -\sum_{i=1}^{N}\left[{\left(\nabla \sigma \right)}_{i}{\hat{y}}_{i}\;\int \;y_{i}^{2}\;\left(\frac{F(r)}{r}\right)\;d^{N}r\right]\\ & = & -\sum_{i=1}^{N}\left[{\left(\nabla \sigma \right)}_{i}{\hat{y}}_{i}\right]\;\int \;\frac{r^{2}}{N}\;\left(\frac{F(r)}{r}\right)\;d^{N}r\\ & = & -\frac{\nabla \sigma }{N}\;\int \;r\;F\left(r\right)\;d^{N}r\;, \end{array}$$

where the $y_{i}$’s stand for the components of the vector $r=(y_{1},\ldots ,y_{N})$, the ${\hat{y}}_{i}$’s are versors (unit vectors) along the coordinate axis, and the ${\left(\nabla \sigma \right)}_{i}$’s are the components of the vector ∇σ. In summary, $F_{\mathrm{int}}$ is given by (6), with

(12) $$D=\frac{1}{N}\int rF\left(r\right)d^{N}r=\frac{\Omega_{N-1}}{N}\int_{0}^{\infty }r^{N}F\left(r\right)dr,$$

where $\Omega_{N-1}$ stands for the total hyper-solid angle associated with an (N−1)-dimensional sphere (i.e., $\Omega_{0}=2$, $\Omega_{1}=2\pi$, and $\Omega_{2}=4\pi$). As we have already seen, the force (11) gives rise to a nonlinear quadratic diffusion term in the evolution Equation (9).

As already explained, the validity of expression (6) for $F_{\mathrm{int}}$ requires the integral $\int_{0}^{\infty }r^{N}F\left(r\right)dr$ to converge. This does not happen if F(r) decreases with *r* more slowly than $r^{-(1+N)}$. We can say that, when deriving expression (6), the criterion used for a power-law force to be a “short-range” one is that it goes as $r^{-\gamma }$ with

(13) γ>1+N.

However, for strict power-law forces satisfying the criterion (13) the expression (6) for $F_{\mathrm{int}}$ is not valid, because the integral $\int_{0}^{\infty }r^{N}F\left(r\right)dr$ diverges due to the singularity of F(r) at r=0. Expression (6) holds for forces F(r) that are short-range (according to the above explained criterion) and that are also well-behaved at r=0.

There are physical scenarios for which it is possible to remove the singularity at r=0 of the integrand of $\int_{0}^{\infty }r^{N}F\left(r\right)dr$, and an expression for $F_{\mathrm{int}}$ similar to (6), but involving higher powers of the particle density can be derived. In those situations, the interaction between the particles can be modeled by a nonlinear power-law diffusion term, as in (9), but with exponents different than 2. These scenarios were discussed in [22] for particles interacting through a force obeying

(14) $$F\left(r\right)=F_{c}\;{\left[\frac{r}{r_{0}}\right]}^{-\gamma },$$

where γ is a positive dimensionless number, and $r_{0}$ and $F_{c}$ are positive constants with dimensions of length and force, respectively. The derivation of (6) implicitly assumes that the typical distance between neighboring particles is small enough so that the lower limit in the integral (the second integral appearing in (12)) defining D can be taken to be r=0. This assumption can be relaxed (see [22,23]), introducing a lower integration cut-off (12)) corresponding to a finite distance $r_{m}>0$. One can imagine that each particle of the system is alone within a small empty sphere of radius $r_{m}$. The effective force acting on a given particle, resulting from interaction with the other particles according to the force law (14), is then $F_{\mathrm{int}}=-D^{*}\nabla \sigma$, with

(15) $$D^{*}=\frac{\Omega_{N-1\;}}{N}\int_{r_{m}}^{\infty }r^{N}F\left(r\right)dr\;,$$

yielding

(16) $$F_{\mathrm{int}}=-\left(\frac{\Omega_{N-1}F_{c}r_{0}^{1+N}}{N(\gamma -N-1)}\right)\;{\left[\frac{r_{m}}{r_{0}}\right]}^{1+N-\gamma }\nabla \sigma .$$

The distance $r_{m}$ is related to the density σ, since the inverse density $\sigma^{-1}$ is proportional to the volume of an *N*-dimensional sphere of radius $r_{m}$. This relation can be cast as $\frac{r_{m}}{r_{0}}={\left(\frac{\sigma }{\sigma_{0}}\right)}^{-1/N}$, where $\sigma_{0}$ is an appropriate constant with dimensions of inverse volume. Finally, using this relation between $r_{m}$ and σ, one can express the force acting on a test particle entirely in terms of the density σ and its gradient,

(17) $$F_{\mathrm{int}}=-\tilde{D}\;\nabla \;\;\left[{\left(\frac{\sigma }{\sigma_{0}}\right)}^{\frac{\gamma -1}{N}}\right],$$

where

(18) $$\tilde{D}=\frac{\Omega_{N-1}F_{c}r_{0}^{1+N}\sigma_{0}}{\left(\gamma -1)(\gamma -N-1\right)}.$$

The spatial density of a system of particles doing overdamped motion, under the effect of the force, evolves according to a nonlinear diffusion equation of the form

(19) $$\frac{\partial \sigma }{\partial t}=D\nabla^{2}\left[\sigma {\left(\frac{\sigma }{\sigma_{0}}\right)}^{1-q}\right]+\nabla \cdot \left[\sigma \nabla V\right],$$

with [22,23],

(20) $$q\;=\;1\;-\;\frac{\gamma -1}{N}.$$

We have re-visited the derivation of nonlinear Fokker–Planck equations for systems of particles interacting via short-range forces, in order to discuss in detail the criterion for “short-range” behind it, which is central to our present work.

## 4. Short-Range versus Long-Range Interactions in Confined Many-Body Systems with Overdamped Motion: A Case Study

The aim of the present section is to discuss a two-dimensional system of confined particles, performing overdamped motion and interacting via *long-range forces*, that exhibits features related to the $S_{q}$-thermostatistics. The time-dependent spatial density of this system complies with an integro-differential evolution equation that admits *q*-Gaussian solutions and evolves in a way similar (but not identical) to the one corresponding to a system governed by a two-dimensional nonlinear power-law Fokker–Planck equation with q=−1. It is instructive to consider the similarities and the differences between the behaviors of these two systems. First we discuss the *q*-Gaussian solutions of the nonlinear Fokker–Planck system, and then the solutions of the system of particles with long-range interactions.

### 4.1. A Two-Dimensional System Described by a Nonlinear Fokker–Planck Equation

We consider the nonlinear Fokker–Planck equation

(21) $$\frac{\partial \sigma }{\partial t}=D\nabla^{2}\left[\sigma {\left(\frac{\sigma }{\sigma_{0}}\right)}^{1-q}\right]+\nabla \cdot \left[\sigma \nabla V\right],$$

where $\sigma_{0}$ and *D* are positive constants, and the confining potential is quadratic,

(22) $$V=\frac{1}{2}c\left(x^{2}+y^{2}\right),$$

with *c* being a positive constant. The evolution Equation (21) admits exact analytical time-dependent solutions of the *q*-Gaussian form,

(23) $$\sigma \left(x,y,t\right)=\sigma_{0}A{\left[1-\left(1-q\right)\beta \left(x^{2}+y^{2}\right)\right]}_{+}^{\frac{1}{1-q}}\;,$$

where the time dependence occurs through the parameters A(t) and β(t). Defining the auxiliary quantity

(24) $$\Gamma ={\left[1-\left(1-q\right)\beta \left(x^{2}+y^{2}\right)\right]}_{+}^{\frac{1}{1-q}},$$

one has

(25) $$\frac{\partial \sigma }{\partial t}=\sigma_{0}\frac{dA}{dt}\Gamma -\sigma_{0}A\frac{d\beta }{dt}\left(x^{2}+y^{2}\right)\Gamma^{q},$$

(26) $$\nabla^{2}\left[\sigma {\left(\frac{\sigma }{\sigma_{0}}\right)}^{1-q}\right]=-4\left(2-q\right)\sigma_{0}A^{2-q}\beta \Gamma +4\left(2-q\right)\sigma_{0}A^{2-q}\beta^{2}\left(x^{2}+y^{2}\right)\Gamma^{q},$$

and

(27) $$\nabla \cdot \left[\sigma \nabla V\right]=2c\sigma_{0}A\Gamma -2c\sigma_{0}A\beta \left(x^{2}+y^{2}\right)\Gamma^{q}.$$

Inserting (25), (26), and (27) into (21), it can be shown that the ansatz (23) constitutes a solution of the evolution Equation (21), provided that the parameters *A* and β satisfy the coupled ordinary differential equations

$$\begin{array}{c} \frac{dA}{dt}=2cA-4\left(2-q\right)DA^{2-q}\beta , \end{array}$$

(28) $$\begin{array}{c} \frac{d\beta }{dt}=2c\beta -4\left(2-q\right)DA^{1-q}\beta^{2}. \end{array}$$

It follows from the above equations that

(29) $$\frac{d}{dt}\left[\frac{\beta }{A}\right]=0,$$

yielding the integration constant

(30) $$B=\frac{\beta }{A}.$$

The evolving density σ(x,y,t) has the norm

(31) $$N\;=\;2\pi \sigma_{0}A\;\int_{0}^{1/\sqrt{2\beta }}\;{\left[1-\left(1-q\right)\beta r^{2}\right]}^{1/2}rdr=\frac{\pi \sigma_{0}}{3}\;\left(\frac{A}{\beta }\right).$$

It is plain that the integral (30) is associated with the preservation of the norm N. Indeed, it follows from the above equation that $N=\pi \sigma_{0}/\left(3B\right)$, from which one sees that the conservation of *B* is equivalent to the conservation of the norm N.

For q=−1 the equations of motion for *A* and β are

$$\begin{array}{ccc} \frac{dA}{dt} & = & 2cA\;-\;12DA^{3}\beta \;, \end{array}$$

(32) $$\begin{array}{ccc} \frac{d\beta }{dt} & = & 2c\beta \;-\;12DA^{2}\beta^{2}\;. \end{array}$$

Using the integral (30), Equations (32) can be written as

$$\begin{array}{ccc} \frac{dA}{dt} & = & 2cA\;-\;12DBA^{4}\;, \end{array}$$

(33) $$\begin{array}{ccc} \frac{d\beta }{dt} & = & 2c\beta \;-\;12DB^{-2}\beta^{4}\;. \end{array}$$

**Figure 1:**
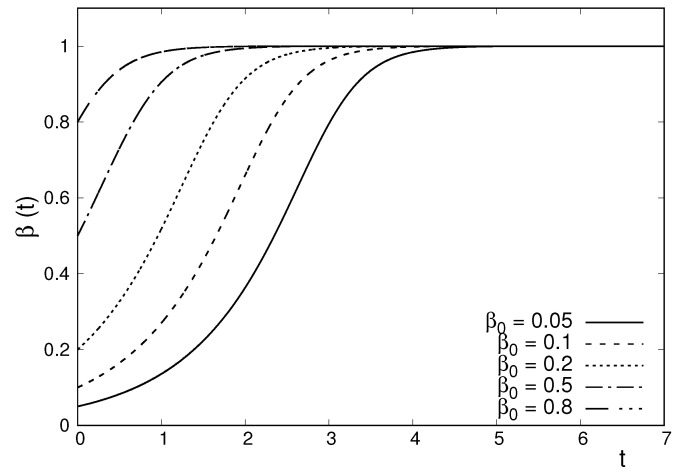
Plot of the time evolution of the parameter β for various initial conditions, corresponding to the *q*-Gaussian solution of a two-dimensional nonlinear Fokker–Planck equation, with q=−1. The parameter evolves according to Equation (33). Units were chosen in such a way that c=1/2, D=1/12, and B=1.

Summing up, the above equations of motion govern a time-dependent *q*-Gaussian density corresponding to a bi-dimensional system with q=−1 that complies with a power-law nonlinear Fokker–Planck evolution equation. According to Equation (20), which relates the space dimension *N*, the entropic index *q*, and the exponent γ characterizing inter-particle forces, the above *q*-Gaussian density can describe the dynamics of a system of particles moving in the overdamped regime, and interacting through short-range forces with γ=5 (i.e., forces behaving with distance as $r^{-5}$). In Figure 1 one can see the evolution of the parameter β, characterizing the width of the *q*-Gaussian for different initial conditions. The parameter β(t) evolves towards the value corresponding to the stationary solution of the nonlinear Fokker–Planck equation.

### 4.2. A Two-Dimensional System with Particles Interacting Through Inverse Square Forces

Now we consider a confined system of particles in the overdamped motion regime that interact through long-range forces. The interactions are via repulsive inverse square forces. The interaction potential between two particles respectively located at r and $r^{\prime }$ is given by $G/|r-r^{\prime }|$, where G is a positive constant. The corresponding force acting on the particle located at x is $G\frac{r-r^{\prime }}{|r-r^{\prime }|3}$. The potential function generated by a planar system of particles, distributed according to the density σ(x,y), is

(34) $$\Phi \left(x,y\right)=G\int \frac{\sigma \left(x^{\prime },y^{\prime }\right)\;dx^{\prime }\;dy^{\prime }}{\sqrt{{\left(x-x^{\prime }\right)}^{2}+{\left(y-y^{\prime }\right)}^{2}}}.$$

Note that the inverse square force law considered here is (except for its sign) mathematically similar to gravitation in three (not two) spatial dimensions. Consequently, our system, in spite of being two-dimensional, has interactions that are similar to those of planar self-gravitational systems embedded in three-dimensional space, such as the planar galaxy models considered in astrophysics [44].

In the regime of overdamped motion, the equation of motion of a test particle moving under the effect of the force due to the potential (34) (due to the interaction with the other particles of the system) and the external confining potential *W* is, according to (5),

(35) $$\frac{dx}{dt}=\frac{1}{\alpha }\left(F_{\mathrm{int}}-\nabla W\right),$$

where

(36) $$F_{\mathrm{int}}=-\nabla \Phi .$$

That is, we have

(37) $$\frac{dx}{dt}=-\frac{1}{\alpha }\left(\nabla \Phi +\nabla W\right).$$

The time evolution of the density σ is then governed by the continuity equation

(38) $$\frac{\partial \sigma }{\partial t}=\frac{1}{\alpha }\nabla \cdot \left[\sigma \nabla \Phi \right]+\nabla \cdot \left[\sigma \nabla V\right],$$

where

(39) $$V\left(x\right)=\frac{1}{\alpha }W\left(x\right).$$

It is important to realize that, in spite of looking like a linear differential equation on the density σ, the evolution Equation (38) is actually a nonlinear integro-differential equation on σ, because the potential function Φ appearing in (38) depends on the density σ through the the relation (34). Indeed, Equation (38) is an instance of an equation of the form

(40) $$\frac{\partial \sigma }{\partial t}=\frac{1}{\alpha }\nabla \cdot \left[\sigma \;\nabla_{x}\int \;\sigma \;U\left(x,x^{\prime }\right)\;d^{N}x^{\prime }\right]+\nabla \cdot \left[\sigma \nabla V\right],$$

with N=2 and $U\left(x,x^{\prime }\right)=G/\left|x-x^{\prime }\right|$. It is worth mentioning that the nonlinear Fokker–Planck Equation (19), when regarded as governing the dynamics of a set of interacting particles, also arises from an evolution equation of the form (40). This happens when the short-range nature of the interactions between the particles makes it possible to transform the integro-differential term in (40) into a purely local differential term.

It is possible to prove, after some algebra, that the *q*-Gaussian ansatz,

(41) $$\sigma \left(x,y\right)=A{\left[1-\left(1-q\right)\beta \left(x^{2}+y^{2}\right)\right]}^{\frac{1}{1-q}},$$

provides exact time-dependent solutions of the evolution Equation (38). In order to prove this, one has to use the fact that the potential Φ generated by the *q*-Gaussian density (41) and Equation (34) is

(42) $$\Phi \left(x,y,\right)=\frac{\pi^{2}\;G\;A}{2\sqrt{2\beta }}\;+\;\frac{\pi^{2}}{2\sqrt{2}}\;G\;A\;\sqrt{\beta }\;\left(x^{2}+y^{2}\right).$$

Details of this calculation can be found in [44], where the gravitational potentials for a family of planar models of disk galaxies are obtained. Except for the minus sign associated with the attractive nature of Newtonian gravitation, the problem of deriving the gravitational potential generated by a planar galaxy model of given surface mass density is mathematically the same as the problem of obtaining, via Equation (34), the potential Φ(x,y) generated by the density σ(x,y).

Inserting (42) in (38), and following a procedure similar to the one adopted in our previous discussion of the solutions of the nonlinear Fokker–Planck equation, one can show that (41) satisfies the evolution Equation (38) if the parameters A and β evolve according to

$$\begin{array}{ccc} \frac{dA}{dt} & = & 2cA\;-\;\sqrt{2}\pi^{2}D\;A^{2}\;\sqrt{\beta }, \end{array}$$

(43) $$\begin{array}{ccc} \frac{d\beta }{dt} & = & 2c\beta \;-\;\sqrt{2}\pi^{2}D\;A\;\beta^{3/2}, \end{array}$$

where $D=\frac{G}{\alpha }$. The above equations of motion admit the integration constant

(44) $$B=\frac{\beta }{A}\;,$$

which can be used to re-cast the equations as

$$\begin{array}{ccc} \frac{dA}{dt} & = & 2cA\;-\;\sqrt{2}\pi^{2}\;D\;\sqrt{B}\;A^{5/2}, \end{array}$$

(45) $$\begin{array}{ccc} \frac{d\beta }{dt} & = & 2c\beta \;-\;\sqrt{2}\pi^{2}\;\frac{D}{B}\;\beta^{5/2}. \end{array}$$

**Figure 2:**
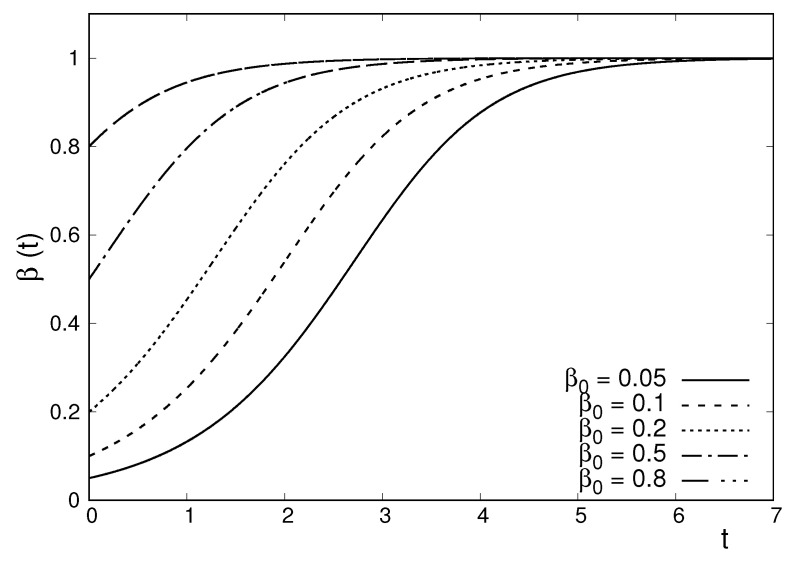
Plot of the time evolution of the parameter β for various initial conditions corresponding to the *q*-Gaussian solution of a two-dimensional system of particles, interacting through inverse square forces and performing overdamped motion. The parameter evolves according to Equation (45). Units were chosen such that c=1/2, $D=1/(\sqrt{2}\pi^{2})$, and B=1.

In Figure 2 we plotted the evolution of the parameter β, characterizing the width of the *q*-Gaussian solution of the integro-differential Equation (38) for different initial conditions. The parameter β(t) evolves towards the value corresponding to the stationary solution of the evolution Equation (38), for which the long-range repulsive forces between the particles are equilibrated by the forces due to the external confining potential.

Interestingly, the overdamped dynamics of the many-body system with long-range interactions discussed here is described by *q*-Gaussians with compact support (i.e., with q<1). Experience from other scenarios with long-range interactions [1] where signatures on $S_{q}$-thermostatistics have been observed suggests that long-range interactions lead to *q*-Gaussians with long tails (q>1). The system analyzed here indicates that this is not necessarily the case. It is worth stressing that the value of *q* associated to this system, which corresponds to *q*-Gaussians with compact support, is not determined by the external confining potential. Instead, the value of *q* is related to the interaction law between the particles. Indeed, the time-dependent *q*-Gaussian solutions, with the same value of *q*, still exist for a vanishing confining potential. When there is no confining potential, the *q*-Gaussian solutions do not relax to a stationary solution, because there are no confining forces to counter-balance the repulsive forces between the particles. Consequently, the time-dependent *q*-Gaussian densities spread forever, exhibiting a behavior similar to pure diffusion. However, the value of *q* is the same whether or not there is a confining potential. In this regard, the role played by the confining potential in our system with long-range interactions is the same as the role it plays in the systems with short-range interactions studied in [20,21,22].

To finalize this section, it is instructive to provide a brief summary of the main similarities and differences between the two systems considered in this work. Both systems consist of interacting particles moving in the overdamped regime under the effect of an external confining potential. In one system the particles interact through short-range repulsive forces following the $r^{-5}$ power-law. The other system has long-range repulsive forces following the inverse square law. Both systems admit time-dependent *q*-Gaussian solutions with q=−1. The *q*-Gaussian densities associated with this value of *q* have compact support. The *q*-Gaussian solutions corresponding to the two systems evolve in a qualitatively similar (but not identical) way, as illustrated in Figure 1 and Figure 2.

## 5. Conclusions

The $S_{q}$-thermostatistics plays a central role in the statistical physics of various many-particle systems with short-range interactions, in the overdamped motion regime, that can be described by nonlinear power-law Fokker–Planck equations. However, the possible relevance of the $S_{q}$-thermostatistics for similar systems with long-range interactions has remained largely unexplored. As a first step in elucidating this subject, we analyzed a system with long-range inverse square repulsive interactions that exhibits a close relationship with the $S_{q}$-thermostatistics. This system is described by an integro-differential evolution equation that admits exact time-dependent solutions of the *q*-Gaussian form. The presence of these types of distributions is one of the main hallmarks and signatures of $S_{q}$-thermostatistics. The evolution of these *q*-Gaussian solutions is similar, but not identical, to the evolution of the *q*-Gaussian solutions corresponding to a two-dimensional nonlinear power-law Fokker–Planck equation. In both cases, the *q*-Gaussian solutions correspond to an entropic parameter q=−1, and the time-dependent parameters characterizing the *q*-Gaussians evolve according to a similar set of coupled ordinary differential equations. However, these evolution equations for the parameters have power-law nonlinearities with exponents that, in the long-range case, are different from those appearing in the nonlinear Fokker–Planck system.

The main take-home message of the present work is that the system investigated here yields evidence indicating that the connection of the $S_{q}$-based statistics with the thermostatistics of overdamped motion of confined interacting many-body systems is not restricted to systems with short-range interactions. This suggests that there may be other interesting connections between two of the main areas of applications of the $S_{q}$-thermostatistics: systems described by nonlinear Fokker–Plank equations on the one hand, and systems having long-range interactions such as gravitation on the other. Further advances along these or related lines will be welcome.
